# Negligible Impact of Mass Screening and Treatment on Mesoendemic Malaria Transmission at West Timor in Eastern Indonesia: A Cluster-Randomized Trial

**DOI:** 10.1093/cid/ciy231

**Published:** 2018-03-22

**Authors:** Inge Sutanto, Ayleen Kosasih, Iqbal R F Elyazar, Deddy R Simanjuntak, Tri A Larasati, M Sopiyudin Dahlan, Isra Wahid, Ivo Mueller, Cristian Koepfli, Rita Kusriastuti, Asik Surya, Ferdinand J Laihad, William A Hawley, Frank H Collins, J Kevin Baird, Neil F Lobo

**Affiliations:** 1 Department of Parasitology, Faculty of Medicine, University of Indonesia, Indonesia; 2 Eijkman-Oxford Clinical Research Unit, Indonesia; 3 Epidemiologi Indonesia, Jakarta, Indonesia; 4 Department of Parasitology, Faculty of Medicine, University of Hasanudin, Makasar, Indonesia; 5 Population Health and Immunity Division, Walter and Eliza Hall Institute, Melbourne, Victoria, Australia; 6 Communicable Disease Control, Ministry of Health, Jakarta, Indonesia; 7 United Nations Children’s Fund, Jakarta, Indonesia; 8 Eck Institute for Global Health, University of Notre Dame, Indiana; 9 Center for Tropical Medicine and Global Health, Nuffield Department of Medicine, University of Oxford, United Kingdom

**Keywords:** cluster-randomized trial, mass screening and treatment, malaria control, Indonesia

## Abstract

**Background:**

Mass screening and treatment (MST) aims to reduce malaria risk in communities by identifying and treating infected persons without regard to illness.

**Methods:**

A cluster-randomized trial evaluated malaria incidence with and without MST. Clusters were randomized to 3, 2, or no MST interventions: MST3, 6 clusters (156 households/670 individuals); MST2, 5 clusters (89 households/423 individuals); and MST0, 5 clusters (174 households/777 individuals). All clusters completed the study with 14 residents withdrawing. In a cohort of 324 schoolchildren (MST3, n = 124; MST2, n = 57; MST0, n = 143) negative by microscopy at enrollment, we evaluated the incidence density of malaria during 3 months of MST and 3 months following. The MST intervention involved community-wide expert malaria microscopic screening and standard therapy with dihydroartemisinin-piperaquine and primaquine for glucose-6 phosphate dehydrogenase–normal subjects. All blood examinations included polymerase chain reaction assays, which did not guide on-site treatment.

**Results:**

The risk ratios for incidence density of microscopically patent malaria in MST3 or MST2 relative to that in MST0 clusters were 1.00 (95% confidence interval [CI], .53–1.91) and 1.22 (95% CI, .42–3.55), respectively. Similar results were obtained with molecular analysis and species-specific (*P. falciparum* and *P. vivax*) infections. Microscopically subpatent, untreated infections accounted for 72% of those infected.

**Conclusions:**

Two or 3 rounds of MST within 3 months did not impact the force of anopheline mosquito-borne infection in these communities. The high rate of untreated microscopically subpatent infections likely explains the observed poor impact.

**Clinical Trials Registration:**

NCT01878357.

## Teaser

This cluster-randomized trial demonstrated a negligible impact of mass microscopic screening in guiding treatment as a means to reduce malaria transmission at a mesoendemic site in Indonesia. Retrospective polymerase chain reaction analysis revealed that the majority of infections were microscopically subpatent.

The World Health Organization recommends mass screening and treatment (MST) as a malaria intervention [1]. MST uses blood samples from all willing residents of endemic communities for diagnostic assessment and treatment of those infected. This strategy targets asymptomatic malaria toward reducing prevalence and continued incidence [2–4]. Minimal impacts of MST on prevalence and incidence have been reported from high-transmission African settings involving *Plasmodium falciparum* [5–11].

Factors driving MST success include achievable coverage and screening technology diagnostic threshold [5–11]. Rapid diagnostic tests (RDTs), an immunochromatographic test, were most commonly used in studies evaluating MST, and reportedly had sensitivity and specificity similar to competent microscopy [12]. The reach of diagnostics directly bears on the coverage issue—that is, the proportion of infected residents cleared of infection [5–11]. Highly sensitive but field-impractical polymerase chain reaction (PCR) is the diagnostic ideal in guiding treatment [13]. Loop-mediated isothermal amplification (LAMP) is a field-adapted molecular diagnostic technology, but is less sensitive than PCR [14].

The timing and frequency of screening, therapies applied, species involved, and intensity of transmission also impact MST. In the Asia Pacific region, relatively lower levels of *P. falciparum* transmission prevail with *Plasmodium vivax* [15]. This study offers a first evaluation of MST in a low-transmission area with *P. falciparum* and *P. vivax*. Antimalarial therapy limited to blood schizontocides includes artemisinin derivatives and partner drugs, which do not impact the latent reservoir of *P. vivax* hepatic hypnozoites [16, 17]. In this cluster-randomized study, MST consisted of expert microscopic mass blood screening guiding the immediate administration of blood schizontocidal therapy together with hypnozoitocidal primaquine for patients diagnosed with *P. vivax*, and gametocytocidal therapy for those having active *P. falciparum* malaria. Control clusters received no MST interventions. We aimed to maximize MST impacts in assessing broader relevance where low-level transmission of both dominant *Plasmodium* species occurs.

## METHODS

### Study Design and Location

**Figure 1.**
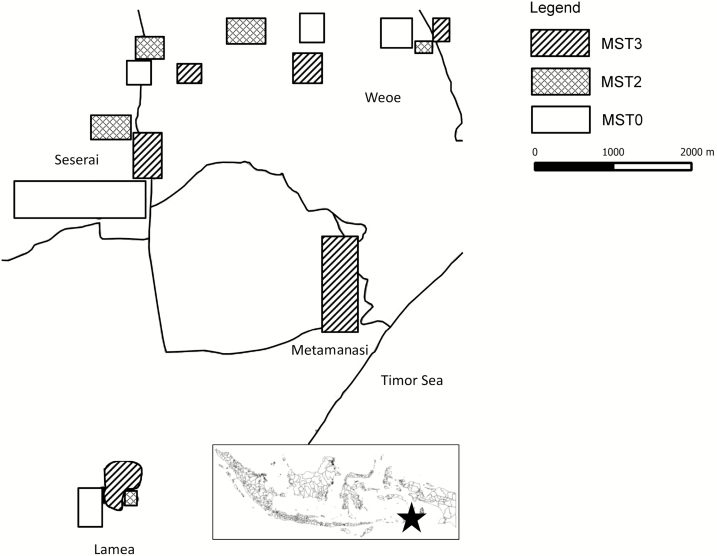
Map of the study site. Abbreviation: MST, mass screening and treatment.

**Figure 2.**
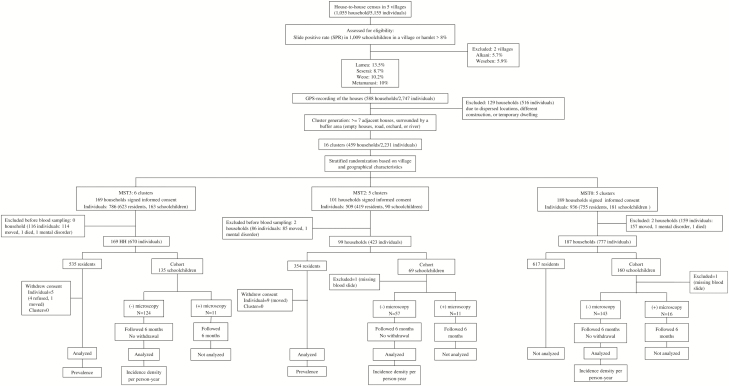
Trial profile. Abbreviations: GPS, Global Positioning System; MST, mass screening and treatment.

Malaria in communities tends toward uneven distributions due to environmental, demographic, and socioeconomic factors [18–20]. This study, conducted during 2013, utilized an open-label, community-wide cluster-randomized controlled trial in Wewiku subdistrict, West Timor, Indonesia (Figure 1). This subdistrict comprised 12 villages having 17423 residents living in traditional coconut palm homes without electrical supply. A tropical climate occurs with a brief wet season (December–March) and extended dry season (April–November). The study coincided with peak malaria transmission during August to September. Annual parasite incidence was 72 and 124 per 1000 person-years during 2011 and 2012, respectively (Belu District Health Office, personal communication). Temperature ranges between 27°C and 35°C with average annual rainfall typically >700 mm. This lowland area (<150 m) includes coastal, savannah, paddy, and forest plantation ecosystems. Ditches, small streams, and semipermanent ground pools are primary anopheline larval habitats. *Anopheles barbirostris* dominates among suspected vector species, followed by *Anopheles subpictus* and *Anopheles vagus*. Limited government vector control during this study included the distribution of 800 insecticide-treated nets during 2011 (Belu District Health Office, personal communication). Because malaria prevalence was highest among coastal villages (Belu District Health Office, personal communication), 5 were selected for screening for inclusion in this study: Alkani, Lamea, Weoe, Seserai, and Weseben (Figure 2).

### Participants

A house-to-house census of the 5 villages occurred in February and April 2013. A census of 8 schools confirmed the names and primary residences of elementary school students, with the baseline malaria screening of 1009 schoolchildren occurring in May 2013. Slide positivity rate (SPR) ranged from 5.7% to 12.7%, indicating mesoendemic transmission [21]. The villages of Lamea, Seserai, Weoe, and the hamlet of Metamanasi (Figure 2), with SPR >8%, were selected as study sites. Households (n = 588) were geolocated and mapped using ArcGIS version 9.3 (Esri, Redlands, California). After excluding 129 households (for isolation, construction, or emptiness; Figure 2), 459 households were assigned to clusters for random allocation to 1 of 3 treatments, that is, MST3:MST2: MST0 (6:5:5). A further 14 households (2 each from MST2 and MST0) were excluded (due to migration, mental illness, or death) prior to the intervention, leaving 455 households (99.1%) with residents participating in the study (Figure 2). The 6, 5, and 5 clusters for 3 rounds of mass screening and treatment (MST3), 2 rounds (MST2), or no intervention (MST0) were home to 670, 423, and 777 residents, respectively. These included a schoolchildren cohort (135, 68, and 159 subjects for MST3, MST2, and MST0 arms, respectively) recruited toward incidence density measurement (Figure 2).

### Procedures

Randomization, intervention allocations and study procedures were explained to community leaders and household heads during a study socialization event. Household heads (n = 459) signed informed consent on behalf of their respective household members. MST3 intervention occurred monthly from June to August 2013, at 5-week intervals. MST2 occurred over the same period with a 10-week interval. MST0 residents were not screened. Schoolchildren cohorts were screened monthly from June to November 2013.

Malaria treatment was administered to all microscopy-positive subjects. Blood (10 μL) from positive individuals was field- examined for glucose-6 phosphate dehydrogenase (G6PD) levels (Trinity Biotech qualitative test). Treatment times, driven by microscopy workload and G6PD assays, ranged from 2 to 3 days after blood collection. Study drugs were temperature monitored (28°C–32°C) and stored in locked cabinets. Drugs included 3-day DHP (fixed-dose tablets of 40 mg dihydroartemisinin, 320 mg piperaquine; D-ARTEPP, Guilin Pharmaceutical Co, China, 6 December 2014 expiry) for all *Plasmodium* species, and primaquine (15-mg primaquine base tablets; PT Phapros Tbk, Jakarta, Indonesia, October 2014 expiry) for *P. falciparum* (single dose of 0.75 mg/kg) or *P. vivax* (daily dose of 0.25 mg/kg over 14 days) in accordance with guidance from the Ministry of Health, Republic of Indonesia [22]. The DHP regimen was 4, 3, 2, 1.5, 1, .5 and .25 tablet(s) daily for patients weighing ≥60 kg, 41–59 kg, 31–40 kg, 18–30 kg, 11–17 kg, 6–10 kg, and ≤5 kg, respectively. For *P. falciparum*, primaquine was given as a single dose on day 1 consisting of 3, 2, 2, 1.5, and .75 tablet(s) for subjects weighing as above, but >10 kg. For *P. vivax* infection the primaquine dose was 1, 1, .75, .5, and .25 tablet(s), as per above weight classes. Primaquine was not administered to 12 residents with abnormal G6PD, 4 infants, 2 underweight children, and 7 *Plasmodium malariae* cases.

Treatment doses and adverse event (AE) monitoring was directly observed by staff at community health centers, health workers at integrated services posts, or elementary school teachers. Drug adherence was defined as taken completely as prescribed with witnessing, and occurred with >90% of cases. Local health centers provided treatment for alternative drugs (eg, quinine for pregnant women) or antipyretics, antiemetics, and antibiotics for AEs and other conditions. All treatments and AEs were reported and recorded daily to the research team.

### Blood Examinations

Finger-prick blood films on glass slides were collected at arranged times and places. Approximately 250 µL of blood was collected in ethylenediaminetetraacetic acid tubes for downstream analyses and stored at −20°C. Thick and thin blood smears were stained 40 minutes with 3% Giemsa and examined using standard oil immersion light microscopy. Parasites were counted against 200 leukocytes and expressed per microliter assuming a leukocyte count of 8000/µL. One hundred ocular fields were examined on site before declaring a smear negative. Agreement between the field and laboratory reader was 0.75. These readings were blinded.

DNA was extracted from whole blood using High Pure PCR Template Preparation kit (Roche Diagnostic) as per the manufacturer’s instruction. The real-time PCR multiplex assay employing SYBR green used a Light Cycler Nano instrument to amplify the 18S ribosomal RNA gene [23]. Details on laboratory procedures are shown in the Supplementary Materials.

### Statistical Analysis

The recruitment target was 1029 subjects per arm, considering a cluster design effect of 1.5, 5% significance, 80% power, and a 1:1 sample size ratio between intervention and control arms. A target of 115 children per arm would yield a power of 82% in detecting an estimated 50% reduction in malaria incidence following MST.

All analyses were performed using IBM SPSS Statistics for Windows version 23 (IBM, Armonk, New York). Change in prevalence was the primary endpoint, examined by comparing prevalence between intervention and control arms using generalized estimating equation modeling for cluster level and the χ^2^ test for individual level [24].

Another primary endpoint was the risk ratio (RR) of *P. falciparum* or *P. vivax* incidence density between the MST3, MST2, and MST0 arms. Incidence was estimated by first microscopic infection during 6 months of observation of schoolchildren. Schoolchildren having microscopically patent parasitemia at enrollment were not analyzed, leaving 124, 57, and 143 malaria-negative children (MST3, MST2, and MST0, respectively; Figure 2). No significant differences were seen in demographic characteristics between 38 malaria-positive (excluded) and 324 malaria-negative (included) children. To adjust for possible clustering effect, the geometric mean of the cluster incidence RR was used to estimate intervention effect [25]. Cox proportional hazards regression calculated the hazard ratio of the individual-level incidence density between the intervention and control arms [26].

Ethical approval was obtained from the Health Research Ethics Committee, Faculty of Medicine, University of Indonesia Cipto Mangunkusumo Hospital (number 39/ H2.FI/ ETHICS/2013) in Jakarta, Indonesia. As the clinical trial authority, inspection by Indonesian Food and Drug Administration was conducted (October 2013).

This study was registered at ClinicalTrials.gov (identifier NCT01878357, June 2013.

## RESULTS

### Study Participants

Similar demographic characteristics occurred among both the cluster and individual levels among MST3, MST2, and MST0 residents (Table 1). Fourteen community residents withdrew (Figure 2), 5 from MST3 and 9 from MST2, due to migration or illness.

**Table 1.** Baseline Characteristics and Blood Sampling Coverage of the Residents and Schoolchildren

Variable	MST3	MST2	MST0	*P*Value^a^
Residents				
Cluster level				
No. of clusters	6	5	5	
Mean No. of households (range)	28 (7–49)	20 (7–39)	37 (8–88)	.516
Mean No. of participants (range)	89 (22–160)	71 (25–139)	124 (22–302)	.586
Mean age, y (range)	31 (28–33)	29 (26–35)	32 (29–38)	.506
Male sex, % (range)	49 (46–53)	52 (44–62)	45 (32–50)	.841
Mean coverage of blood sampling, % (range)	87 (81–90)	89 (86–92)		
Individual level				
Total participants	535	354	617	
No. of households	169	99	187	
Mean age, y (range)	31 (0.1–86)	30 (0.2–96)	31 (0.1–80)	.640
Male sex, No. (%)	260 (48.6)	180 (50.7)	285 (46.2)	.393
Mean coverage of blood sampling, % (range)	88 (82–92)	88 (86–91)		
Schoolchildren				
Cluster level				
No. of clusters	6	5	5	
Mean No. of households (range)	14 (2–23)	8 (2–16)	20 (6–50)	.348
Mean participants (range)	21 (4–37)	11 (2–29)	29 (9–74)	.372
Mean age, y (range)	10 (9–11)	9 (7–11)	9 (9–10)	.378
Male sex, % (range)	53 (34–83)	53 (50–58)	52 (33–80)	.324
Mean coverage of blood sampling, % (range)	98 (98–100)	99 (95–100)	96 (94–99)	
Individual level				
Total children	124	57	143	
No. of households	81	41	99	
Mean age, y (range)	10 (6–13)	9 (6–15)	9 (6–14)	.546
Male sex, No. (%)	60 (48.4)	31 (54.4)	67 (46.9)	.626
Mean coverage of blood sampling, % (range)	98 (98–100)	99 (96–100)	97 (96–100)	

Abbreviation: MST, mass screening and treatment.

^a^χ^2^ test for categorical variables, and analysis of variance for numerical variables, except for mean age in residents (Kruskal-Wallis test).

### Community Malaria Prevalence: Cluster and Individual Analysis

#### Microscopic Examinations

**Figure 3.**
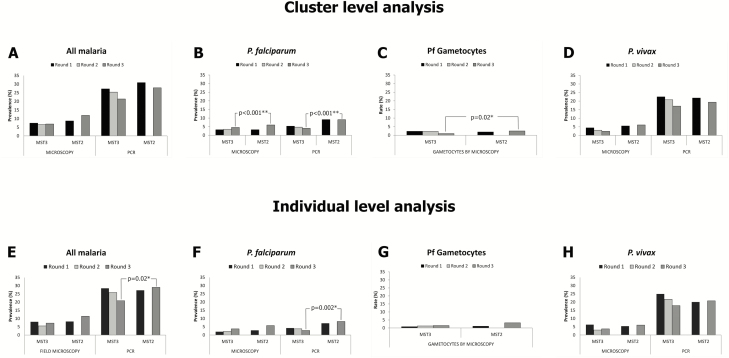
Malaria prevalence by microscopy and polymerase chain reaction (PCR), and *Plasmodium falciparum* gametocyte rate by microscopy at the cluster (*A*–*D*) and individual levels (*E–H*). Cluster level showed significant decrease of *P. falciparum* infections based on microscopic and PCR examinations at the last survey (*B*). Individual level demonstrated this change with PCR (*F*). Similarly, gametocytes of *P. falciparum* that was detected by microscopy demonstrated significant decrease in cluster level (*C*). In *Plasmodium vivax* infections, neither change was detected in the cluster and individual levels (*D* and *H*). The χ^2^ test was used for individual level, and generalizing estimating equations was used for cluster level. Abbreviation: MST, mass screening and treatment.

Microscopic assessments of the first round of MST showed similar SPR between MST3 and MST2: cluster level: 7.4% vs 8.7%, odds ratio [OR], 1.00 (95% confidence interval [CI], .49–2.01), *P* = .993; individual level: 8.1% vs 8.2%, OR, 0.98 (95% CI, .56–1.71), *P* = 1.000 (Figure 3A and 3E). Similar findings emerged in species-specific infections—by *P. falciparum*: cluster level: 3.2% vs 3.2%, OR, 0.93 (95% CI, .39–2.24), *P* = .877; individual level: 2.0% vs 2.9%, OR, 0.70 (95% CI, .26–1.88), *P* = .645 (Figure 3B and 3F) and by *P. vivax*: cluster level: 4.4% vs 5.5%, OR, 1.04 (95% CI, .47–2.30), *P* = .922; individual level: 6.3% vs 5.4%, OR, 1.18 (95% CI, .61–2.29), *P* = .738 (Figure 3D and 3H). Microscopy-based parasitemia and treatment were evenly distributed between MST3 and MST2 clusters.

A significant difference occurred with *P. falciparum* infections (cluster-level analysis) following the last round of MST: 4.6% vs 6.0%, OR, 0.47 (95% CI, .33–.67), *P* < .001. This was not significant in the individual-level analysis: 3.8% vs 5.7%, OR, 0.65 (95% CI, .31–1.33), *P* = .312 (Figure 3B and 3F). Neither cluster- nor individual-level differences were significant in the last MST survey among *P. vivax* infections: cluster level: 2.4% vs 6.1%, OR, 0.49 (95% CI, .15–1.60), *P* = .240; individual level: 3.8% vs 6.1%, OR, 0.61 (95% CI, .30–1.23), *P* = .226 (Figure 3D and 3H).

Preintervention, similar *P. falciparum* microscopy-based gametocyte rates were observed between MST3 and MST2; cluster level: 2.3% vs 1.9%, OR, 1.14 (95% CI, .47–2.76), *P* = .773; individual level: 0.8% vs 1.1%, OR, 0.70 (95% CI, .14–3.50), *P* = .695 (Figure 3C and 3G). However, *P. falciparum* gametocyte rates were significantly lower in MST3 vs MST2 at the last round of MST in the cluster analysis: 1% vs 2.5%, OR, 0.26 (95% CI, .09–0.77), *P* = .015 (Figure 3C). This significance did not occur in individual-level analyses: 1% vs 3.2%, OR, 0.31 (95% CI, .09–1.00), *P* = .075 (Figure 3G).

#### PCR Examinations

Preintervention, the proportion of malaria positives by PCR in MST3 relative to MST2 was not significantly different: cluster level: 27.3% vs 30.9%, OR, 0.98 (95% CI, .66–1.44), *P* = .905; individual level: 28.5% vs 27.2%, OR, 1.06 (95% CI, .75–1.50), *P* = .793 (Figure 3A and 3E). Similarly, no significant differences occurred in *P. falciparum* prevalence: cluster level: 5.3% vs 9.1%, OR, 0.82 (95% CI, .51–1.33), *P* = .418; individual level: 4.3% vs 7.2%, OR, 0.58 (95% CI, .30–1.13), *P* = .146 (Figure 3B and 3F). No differences were seen with *P. vivax* infections: cluster level: 22.5% vs 21.8%, OR, 1.11 (95% CI, .76–1.64), *P* = .581; individual level: 24.9% vs 20.1%, OR, 1.32 (95% CI, .91–1.92), *P* = .165 (Figure 3D and 3H). PCR patent parasitemia was evenly distributed between the MST3 and MST2 clusters before intervention.

At the last intervention survey, both cluster- and individual-level analyses demonstrated significantly lower *P. falciparum* in MST3 relative to MST2: cluster level: 4.1% vs 9.1%, OR, 0.25 (95% CI, .21–.31), *P* < .001; individual level: 2.8% vs 8.2%, OR, 0.32 (95% CI, .15–.66), *P* = .002 (Figure 3B and 3F). These differences were not observed with *P. vivax*: cluster level: 17.1% vs 19.4%, OR, 0.87 (95% CI, .54–1.40), *P* = .569; individual level 17.9% vs 20.8%, OR, 0.83 (95% CI, .56–1.22), *P* = .397 (Figure 3D and 3H). The additional MST round approximately halved *P. falciparum* prevalence but did not impact *P. vivax* prevalence.

### Malaria Incidence Among Cohort Schoolchildren: Cluster and Individual Analysis

#### Microscopic Examinations

**Figure 4.**
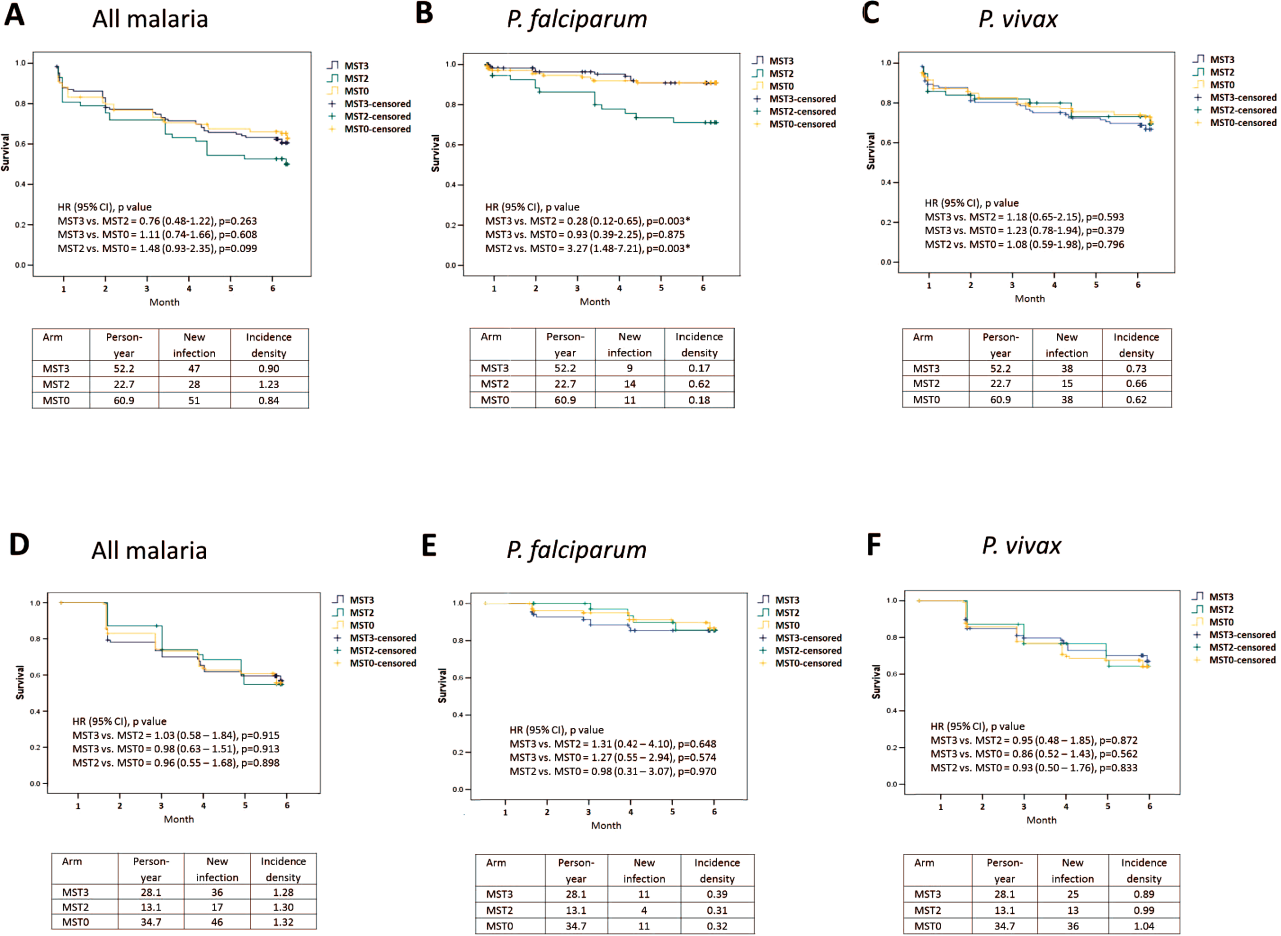
Microscopic (*A–C*) and polymerase chain reaction (*D–F*) based individual malaria incidence density of schoolchildren in all arms within 6 months. No significant difference of incidence density in all malaria, *Plasmodium falciparum*, and *Plasmodium vivax* infections between arms. Hazard ratios were estimated using Cox regression analysis. Abbreviations: CI, confidence interval; HR, hazard ratio; MST, mass screening and treatment.

Based on cluster analysis, malaria incidence densities (first event per person-year) were 1.16, 2.87, and 1.22 for MST3, MST2, and MST0, respectively (Table 2). The 3 groups of schoolchildren showed similar risk of malaria infection: MST3 vs MST2: RR, 1.42 (95% CI, .44–4.60); MST3 vs MST0: RR, 1.00 (95% CI, .53–1.91); MST2 vs MST0: RR, 1.22 (95% CI, .42–3.55). These risks were similar for *P. falciparum*: MST3 vs MST2: RR, 0.68 (95% CI, .19–2.45); MST3 vs MST0: RR, 1.04 (95% CI, .23–4.80); MST2 vs MST0: RR, 1.61 (95% CI, .23–9.36), and for *P. vivax*: MST3 vs MST2: RR, 0.89 (95% CI, .41–1.93); MST3 vs MST0: RR, 0.99 (95% CI, .62–1.59); MST2 vs MST0: RR, 1.04 (95% CI, .36–2.98) (Table 2). Similar results were obtained with individual analyses (Figure 4A–C).

**Table 2.** Incidence Density and Risk Ratio With Cluster Effect in Schoolchildren

Arm	All Malaria	*Plasmodium falciparum*	*Plasmodium vivax*
Microscopic-based			
No. of new infection per cluster (range)			
MST3	7.8 (2–13)	1.5 (0–3)	6.3 (1–11)
MST2	5.6 (2–16)	2.8 (1–6)	3.0 (1–10)
MST0	10.2 (1–20)	2.2 (0–4)	7.6 (1–16)
Incidence density per cluster (range)			
MST3	1.16 (0.71–2.12)	0.47 (0.00–1.31)	0.78 (0.56–1.06)
MST2	2.87 (0.69–10.00)	1.48 (0.46–5.00)	1.44 (0.20–5.00)
MST0	1.22 (0.18–3.02)	0.46 (0.00–1.72)	0.74 (0.18–1.29)
Risk ratio (95% CI)			
MST3 vs MST2	1.42 (.44–4.60)	0.68 (.19–2.45)	0.89 (.41–1.93)
MST3 vs MST0	1.00 (.53–1.91)	1.04 (.23–4.80)	0.99 (.62–1.59)
MST2 vs MST0	1.22 (.42–3.55)	1.61 (.23–9.36)	1.04 (.36–2.98)
PCR-based			
Number of new infection per cluster (range)			
MST3	13.2 (4–26)	2.5 (1–5)	10.8 (2–21)
MST2	8.0 (2–23)	2.0 (0–7)	6.4 (2–17)
MST0	16.4 (1–34)	3.4 (0–6)	13.0 (1–28)
Incidence density per cluster (range)			
MST3	3.03 (1.37–7.39)	1.02 (0.20–3.70)	2.11 (1.17–3.69)
MST2	3.83 (1.10–5.42)	0.90 (0.00–2.44)	3.40 (0.88–5.42)
MST0	2.67 (0.20–6.48)	0.71 (0.00–2.16)	1.96 (0.20–4.32)
Risk ratio (95% CI)			
MST3 vs MST2	0.89 (.43–1.83)	0.82 (.25–2.67)	0.85 (.43–1.68)
MST3 vs MST0	1.24 (.31–4.98)	0.91 (.27–3.10)	1.23 (.34–4.46)
MST2 vs MST0	1.40 (.33–5.98)	1.04 (.33–3.28)	1.44 (.34–6.15)

The table shows no significant difference of cluster-based malaria incidence density of both species among all arms. Risk ratio was calculated from the ratio of incidence density in the intervention and control arms. Geometric mean of cluster incidence was used to adjust the possible clustering effect.

Abbreviations: CI, confidence interval; MST, mass screening and treatment; PCR, polymerase chain reaction.

#### PCR Examinations

Cluster-based incidence density of malaria infections measured by PCR was 3.03, 3.83, and 2.67 infections per person-year for MST3, MST2, and MST0, respectively (Table 2). No significant differences in all malaria infections appeared among the 3 groups: MST3 vs MST2: RR, 0.89 (95% CI, .43–1.83); MST3 vs MST0: RR, 1.24 (95% CI, .31–4.98); MST2 vs MST0: RR, 1.40 (95% CI, .33–5.98) (Table 2). The same was seen in species-specific subanalyses: *P. falciparum*, MST3 vs MST2: RR, 0.82 (95% CI, .25–2.67), MST3 vs MST0: RR, 0.91 (95% CI, .27–3.10), MST2 vs MST0: RR, 1.04 (95% CI, .33–3.28) (Table 2); *P. vivax*, MST3 vs MST2: RR, 0.85 (95% CI, .43–1.68), MST3 vs MST0: RR, 1.23 (95% CI, .34–4.46), MST2 vs MST0: RR, 1.44 (95% CI, .34–6.15) (Table 2). Similar results were obtained with individual analyses (Figure 4D–F).

### Submicroscopic Infections

During MST activities, a total of 2016 blood samples were collected and 26.8% (540) were positive, either by microscopy (21 [3.9%]), PCR (391 [72.4%]), or both (128 [23.7%]). Only 27.6% (149/540) of malaria infections were treated in connection with MST activities. Subpatent microscopic infections (n = 391) consisted of 48 *P. falciparum* (12.2%), 331 *P. vivax* (84.7%), and *P. malariae* (5 [1.3%]) or mixed infections (4 *P. falciparum/P. vivax* [1.0%] and 3 *P. vivax/P. malariae* [0.8%]) (Table 3).

**Table 3.** Proportion of Submicroscopic Malaria Infections

	Total Blood Samples	Infections by Microscopy or PCR	Treated Infections^a^, No. (%)	Untreated Infections^b^, No. (%)	Pf by Microscopy or PCR	Treated Infections^a^, No. (%)	Untreated Infections^b^, No. (%)	Pv by Microscopy or PCR	Treated Infections^a^, No. (%)	Untreated Infections^b^, No. (%)	Pm and Mixed Infection by Microscopy or PCR	Treated Infections^a^, No. (%)	Untreated Infections^b^, No. (%)
MST3													
June	488	141	37 (26)	104 (74)	23	11 (48)	12 (52)	111	23 (21)	88 (79)	7	3 (43)	4 (57)
July	473	124	25 (20)	99 (80)	20	11 (55)	9 (45)	98	11 (11)	87 (89)	6	3 (50)	3 (50)
August	433	94	26 (28)	68 (72)	13	9 (69)	4 (31)	75	15 (20)	60 (80)	6	2 (33)	4 (66)
Subtotal	1394	359	88 (25)	271 (75)	56	31 (55)	25 (45)	284	49 (17)	235 (83)	19	8 (42)	11 (58)
MST2													
June	321	92	25 (27)	67 (73)	21	9 (43)	12 (57)	69	14 (20)	55 (80)	2	2 (100)	0 (0)
August	301	89	36 (40)	53 (60)	28	17 (61)	11 (39)	58	17 (29)	41 (71)	3	2 (67)	1 (33)
Subtotal	622	181	61 (34)	120 (66)	49	26 (53)	23 (47)	127	31 (24)	96 (76)	5	4 (80)	1 (20)
Total	2016	540	149 (28)	391 (72)	105	57 (54)	48 (46)	411	80 (19)	331 (81)	24	12 (50)	12 (50)

Abbreviations: MST, mass screening and treatment; PCR, polymerase chain reaction; Pf, *Plasmodium falciparum*; Pm, *Plasmodium malariae*; Pv, *Plasmodium vivax*.

^a^Number of infections detected by microscopy and treated with antimalarial drugs.

^b^Number of infections detected by PCR that were not treated with antimalarial drugs.

### Adverse Events

Drug administration during this study did not prompt withdrawal of any subject, and no serious AEs occurred. The most common AEs during treatment were fever (0.023/person-day), headache (0.008/person-day), vomiting (0.006/person-day), cough (0.004/person-day), shivering (0.003/person-day), and nasal congestion (0.002/person-day).

## DISCUSSION

This study demonstrated that MST may have little or no impact on malaria transmission in endemic communities where the majority of malaria infections cannot be detected by standard point-of-care diagnostics. Unrestricted malaria infection occurred with 2 or 3 rounds of MST, despite screening coverage >80%—higher than that typically attained in practice. Dominant subpatent and asymptomatic reservoirs, along with the latent hypnozoite reservoir of *P. vivax*, appear to have sustained transmission during the 3 months of intervention and the following 3 months. Thus, despite far lower transmission intensity, expert microscopy, and the use of primaquine as a transmission-blocking gametocytocide and hypnozoitocide, this study demonstrated similar results in the African setting of intense *P. falciparum* transmission diagnosed by RDT and treated with artemisinin-based combination therapy alone [5–11]. Our attempt to optimize microscopy-based MST for an Asian setting thus appears to be proven futile.

A 2-fold statistically significant decrease between the MST3 and MST2 arms in *P. falciparum* prevalence was observed at the last round of MST by both microscopy and PCR diagnosis. This may be attributed to the greater efficiency of diagnosis of *P. falciparum* relative to *P. vivax* (ie, 50% vs 20%; Table 3). Furthermore, the decrease of gametocyte carriers of *P. falciparum* in MST3 relative to MST2 (Figure 3C) did not translate to diminished risk of new infection.

In *P. vivax*–infected subjects, 3 recurrences appeared within 2 months among 80 residents given antirelapse primaquine therapy (3.6%). In the cohort of schoolchildren, 10.5% (9/86) had recurrences within 5 months. All subjects received 0.25 mg/kg per national treatment guidelines; this relatively low dose may be inadequate [27]. The significant effort and expense made to safely diminish hypnozoite-borne blood infection in these communities with primaquine therapy exerted no discernible impact on risk of acute patent *P. vivax*, likely due to the inability to diagnose and treat the majority of subpatent infections. The latent reservoir of vivax malaria was not significantly impacted by MST that included a hypnozoitocide.

There are several important limitations to this study. Presumptive radical cure of the schoolchildren cohorts—to eliminate subpatent malaria infections—did not occur. These preinfections, rather than biting infectious mosquitoes, may account for some of the new infections detected and counted. Nonetheless, these were assumed to be balanced among the randomized arms, and therefore minimally impacting the MST effectiveness endpoints of RR. Another weakness may be the relatively small number of clusters per MST intervention (5 or 6), thus limiting the statistical power (<80%) to detect relatively small differences. Subtle but real differences are unimportant in the context of practice, where impacts must be relatively large for justifying costly and labor-intensive MST operations.

Additionally, the enrollment targets based on detecting a 50% reduction were not met. The RR near unity pointed to little difference of impact between arms, and the proportionate CI supported this finding (Table 2). Thus, this negative result was not likely caused by the small sample size [28, 29]. The shortfall in sample size did not statistically impede the assessments.

In summary, 2 or 3 rounds of MST had no impact on the force of malaria infections in these communities. Effective MST requires improved sensitivity in point-of-care diagnostics [30]. Both highly sensitive antigen-detecting RDTs and near-patient malaria LAMP are now commercially available [14, 31]. These tools may measurably improve the efficacy of MST [14, 30]. Additionally, combining MST with other interventions that minimizing human–mosquito contact, may demonstrate detectable impacts. Finally, MST undertaken here exploited the availability of point-of-care diagnostics for G6PD deficiency and included hypnozoitocidal therapy for an underdiagnosed, understudied, and undertreated clinical and public health problem [32–35]. While we demonstrated the operational feasibility of safely including hypnozoitocidal primaquine therapy, inadequate infection diagnostics diminished possible impacts.

## Supplementary Data

Supplementary materials are available at *Clinical Infectious Diseases* online. Consisting of data provided by the authors to benefit the reader, the posted materials are not copyedited and are the sole responsibility of the authors, so questions or comments should be addressed to the corresponding author.

Supplementary Materials

## Notes

***Acknowledgments.*** We thank Theresia M. B. Saik, Siprianus Mali, and Fransiska Funan in the Belu Regency Health Office for their support and cooperation; we also thank Brigitha Bria, and staff of the Weoe Health Center, for their invaluable assistance during the study. Furthermore, we owe a debt of gratitude to Lina Sembiring and staff of Betun Hospital, and teachers and staff from elementary schools in Wewiku for their support and participation during the study. We give sincere gratitude to Sri Suprijanto, Rosidi Rayhan, and Budi Prasetyorini for their excellent microscopic reading, and field team members and laboratory officers (Cathy Wabang, Emerensiana Meno, Maria Bouk, Imelda Goncales, Juan Selan, Mariana Fatuli, Dorothea Niron, Juleta Matmof, and George Mata) for their dedicated work. We also appreciate the outstanding work from laboratory staff in the Eijkman-Oxford Clinical Research Unit (Decy Subekti, Jeny, Lia Waslia, Damian A. Oyong, and Saraswati Soebianto). We acknowledge Hengki Tasman for conducting census and mapping in the study site. We are also grateful to PT Bhineka Usada Raya for supplying the antimalarial drug D-ARTEPP, and PT Phaphros Tbk for supplying primaquine. We are also indebted to John E. Gimnig (Centers for Disease Control and Prevention, Atlanta, Georgia) for his assistance in study design; Ermi Ndoen (UNICEF Kupang) for his support during the study; and former Dean of Medical Faculty, University of Indonesia, Ratna Sitompul and former Research Manager of Medical Faculty, University of Indonesia, Ponco Birowo, for their great support to make this study possible. Finally, we thank all study participants, cadres, and the community leaders for their immeasurable support during the study.

***Financial support.*** This project has been funded in whole or in part with funds from the Bill & Melinda Gates Foundations (grant number 45114).

***Potential conflicts of interest.*** All authors: No reported conflicts of interest. All authors have submitted the ICMJE Form for Disclosure of Potential Conflicts of Interest. Conflicts that the editors consider relevant to the content of the manuscript have been disclosed.
