# Supplementary material for: Negligible Impact of Mass Screening and Treatment on Mesoendemic Malaria Transmission at West Timor in Eastern Indonesia: A Cluster-Randomized Trial
Source: Clin Infect Dis. 2018 Mar 22;67(9):1364–72. doi: 10.1093/cid/ciy231 (PMC6186863; doi:10.1093/cid/ciy231)
Supplement: Supplementary Materials [file ciy231_suppl_supplementary_file_1.docx]

**Supplementary File 1**

**Laboratory Procedure**

**Microscopic Examination**

Finger pricked blood samples for microscopic slide exams were collected from MST3 and MST2 residents during each round of MST. Similar samples were also taken monthly from schoolchildren during MST3, MST2, and MST0. Additionally, ~250 µL finger pricked whole blood from was collected in EDTA tubes for downstream analyses - including PCR. Thick and thin blood smears were stained for 40 minutes with 3% Giemsa. Plasmodium parasites were counted against 200 leukocytes and expressed per microliter assuming a leukocyte count of 8,000/µL. One hundred ocular fields were examined before declaring a smear negative. The first reading was performed in the field and the second reading (blinded to the first reading) in the central laboratory in Jakarta. In the case of a disagreement, another microscopist re-read the slide and the final confirmed results were based on the last reader. Agreement between the field and laboratory reader were 0.75. All microscopists were WHO certified as ‘advanced’ (1 person) and ‘experts’ (3 persons).

**Real-Time PCR**

DNA extraction was performed at the Eijkman-Oxford Clinical Research Unit (EOCRU) laboratory in Jakarta using high pure PCR template preparation kit (Roche Diagnostics) in accordance to manufacturer’s instruction, with an addition of 200 µL of PBS into each sample prior to extraction. The DNA extracted from 200 µL blood was eluted with 100 µL of elution buffer.

Multiplexed, SYBR green based, Real-Time PCR (1) was performed in a LightCycler Nano (Roche Diagnostics) in the Pharmacology laboratory of the Medical Faculty, University of Indonesia. The multicopy 18S rRNA gene was targetted using the forward primer (5’- TAA CGA ACG AGA TCT TAA-3’) and reverse primer (was 5’- GTT CCT CTA AGA AGC TTT-3’) (1). The reaction was performed in a 20 uL mix containing 1x FastStart Essential DNA Green Master (Roche Diagnostics), 0.5 µM of each primer, and 5 uL of DNA template with condition as follows: initial denaturation 95^o^C for 10 minutes, followed by 45 cycles of denaturation at 95^o^C for 10 seconds, annealing at 48^o^C for 10 seconds, and extension at 72^o^C for 20 seconds, and melting analysis by stepwise temperature increase at 0,1^o^C/second from 60^o^C to 95^o^C. Samples demonstrating amplification curve (Figure S1) with species-specific Tm value were deemed positive for malaria. Plasmodium species was determined by the melting temperature (Tm) value - which was slighly different from that of the reference. A T_M_ value between 72.0-73.5 was found to indicate *P. malariae*, a Tm value between 73.5-75.0 indicated *P. falciparum*, and a Tm value between 76.5-78.5 determined *P. vivax* (Figure S2). These control values were obtained from positive controls. An evaluation of the assay using serial dilutions of known positive samples run in triplicate (*P. falciparum* 3,338/µL, *P. vivax* 48 parasites/µL, *P. malariae* 340 parasites/µL) determined the reaction sensitivity to be 2, 1, and 5 parasites/µL for *P. falciparum*, *P. vivax* and *P. malariae* respectively. Positive controls for *P. falciparum* and *P. vivax* (each 3 parasites/µL), as well as *P. malariae* (5 parasites/µL) were included in each run as well as negative control which consisted of no template control (NTC) and DNA of uninfected blood.


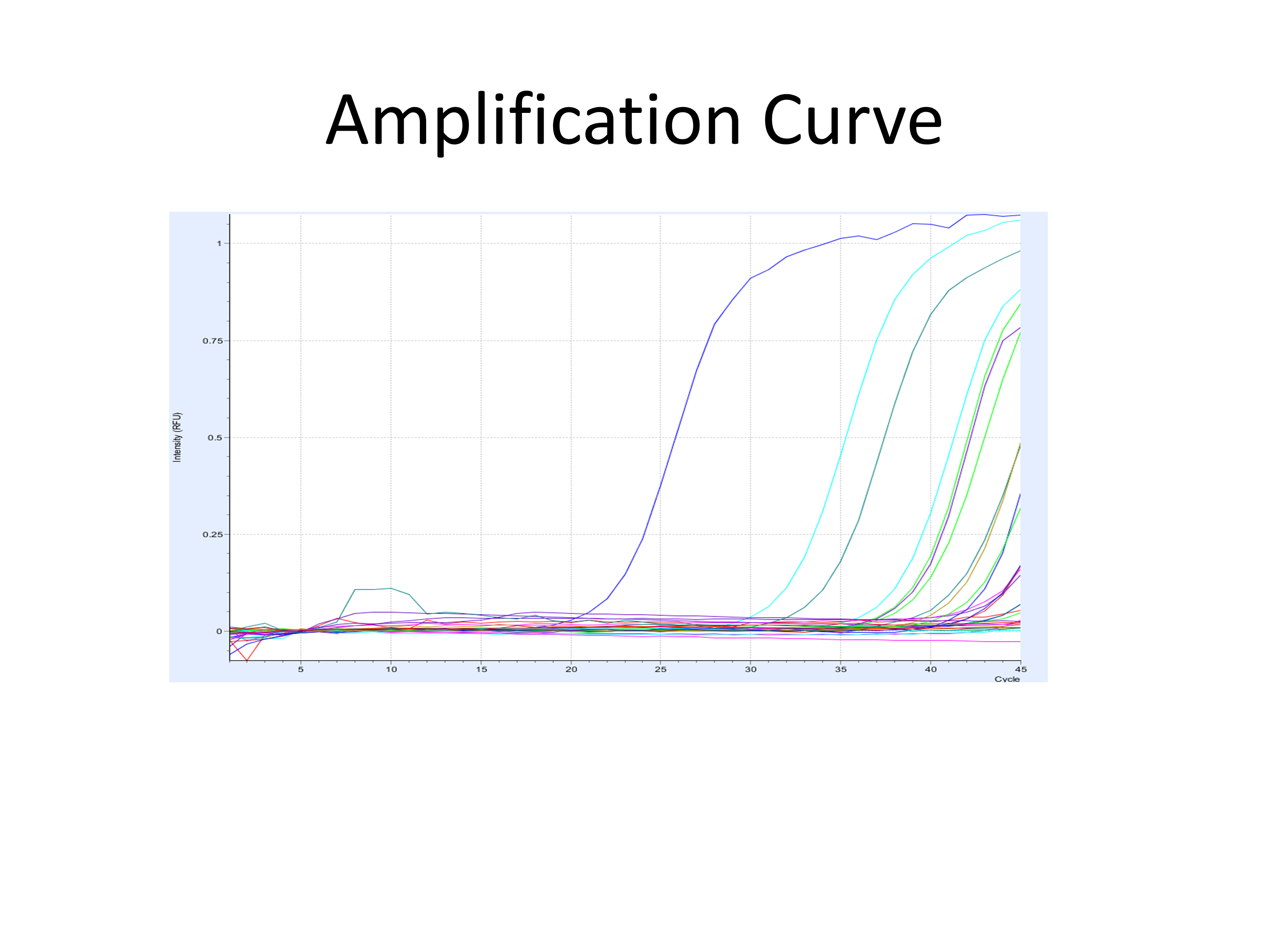


Figure S1. Amplification curve of all samples in one run.


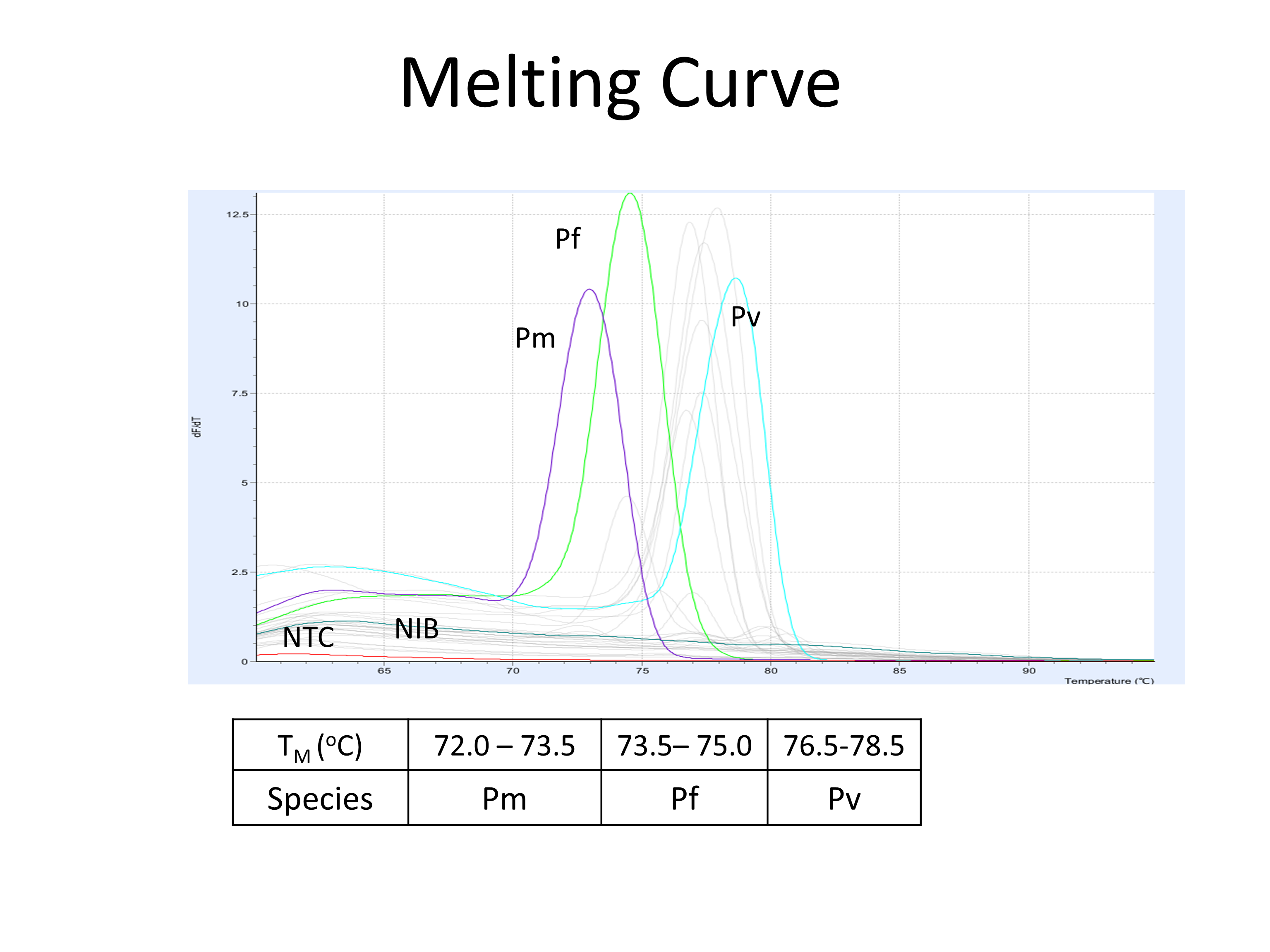


Figure S2. T_M_ value of the *Plasmodium* species. Abbreviation: Pf - *Plasmodium falciparum*, Pv - *Plasmodium vivax*; Pm - *Plasmodium malariae*; NTC - no template control; NIB - non infected blood

References

1. Mangold K, Manson R, Koay E, Stephens L, Regner M, Thomson J. Real-time PCR for detection and identification of Plasmodium spp. J Clin Microbiol. 2005;43(5):2435–40.
